# Polymer Functionalized Nanoparticles in Blue Phase LC: Effect of Particle Shape

**DOI:** 10.3390/nano12010091

**Published:** 2021-12-29

**Authors:** Manlin Zhang, Michael Lindner-D’Addario, Mahdi Roohnikan, Violeta Toader, Robert Bruce Lennox, Linda Reven

**Affiliations:** Centre Québécois sur les Matériaux Fonctionnels/Quebec Centre for Advanced Materials (CQMF/QCAM), Department of Chemistry, McGill University, Montreal, QC H3A 0B8, Canada; Manlin.Zhang@mail.mcgill.ca (M.Z.); michael.lindner-daddario@mail.mcgill.ca (M.L.-D.); Mahdi.Roohnikan@mail.mcgill.ca (M.R.); Violeta.Toader@mcgill.ca (V.T.); bruce.lennox@mcgill.ca (R.B.L.)

**Keywords:** blue phase liquid crystals, gold nanoparticles, polymers

## Abstract

Ethylene oxide oligomers and polymers, free and tethered to gold nanoparticles, were dispersed in blue phase liquid crystals (BPLC). Gold nanospheres (AuNPs) and nanorods (AuNRs) were functionalized with thiolated ethylene oxide ligands with molecular weights ranging from 200 to 5000 g/mol. The BPLC mixture (ΔT_BP_ ~6 °C) was based on the mesogenic acid heterodimers, n-hexylbenzoic acid (6BA) and n-*trans*-butylcyclohexylcarboxylic acid (4-BCHA) with the chiral dopant (R)-2-octyl 4-[4-(hexyloxy)benzoyloxy]benzoate. The lowest molecular weight oligomer lowered and widened the BP range but adding AuNPs functionalized with the same ligand had little effect. Higher concentrations or molecular weights of the ligands, free or tethered to the AuNPs, completely destabilized the BP. Mini-AuNRs functionalized with the same ligands lowered and widened the BP temperature range with longer mini-AuNRs having a larger effect. In contrast to the AuNPs, the mini-AuNRs with the higher molecular weight ligands widened rather than destabilized the BP, though the lowest MW ligand yielded the largest BP range, (ΔT_BP_ > 13 °C). The different effects on the BP may be due to the AuNPs accumulating at singular defect sites whereas the mini-AuNRs, with diameters smaller than that of the disclination lines, can more efficiently fill in the BP defects.

## 1. Introduction

**Figure 1:**
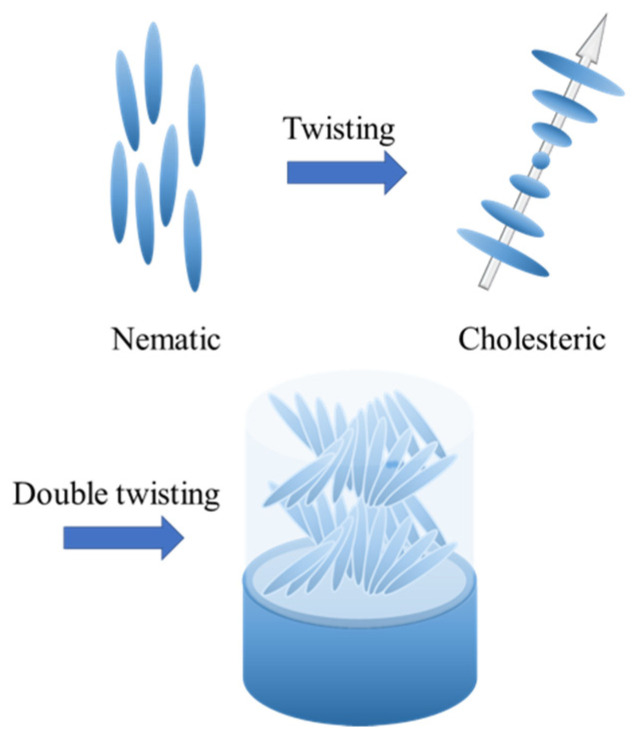
Schematic of the cholesteric and double-twist cylinder.

**Figure 2:**
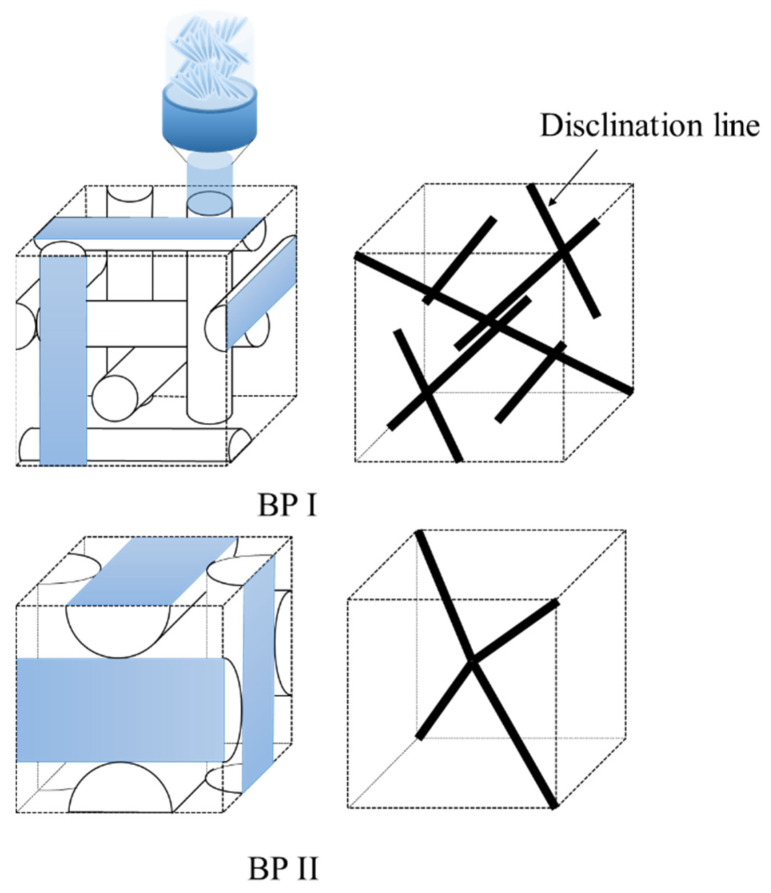
A schematic of the BP I and BP II structures showing the packing of the double-twist cylinders along with the corresponding defect lattice. Adapted with permission from [8]. Copyright RSC Publishing, 2013.

Cholesteric blue phase liquid crystals (BPLCs) are chiral liquid crystals with unique cubic structures that have optical isotropy and very fast response times to electrical fields, making them interesting candidates for display and other electro-optical applications [1,2,3,4,5,6,7,8]. Their structure is characterized by a local director field that forms double-twisted supramolecular helicoids (Figure 1). Since the double twisted arrangement cannot perfectly occupy a 3D space, line defects known as twist disclinations are formed, as shown in Figure 2. In other words, the blue phase spontaneously forms an ordered defect structure [5]. The three different structures of BP are characterized by decreasing temperature: these are denoted as BP III, BP II and BP I. BP I self-arranges into body-centered cubic (BCC) and BP II into simple cubic (SC). The “blue fog” (BP III) has an isotropic symmetry and reflects light in a broad manner, which can be difficult to detect due to its extremely narrow temperature range (~0.1 °C) [6]. BP I and BP II appear as multi-color platelets under the polarized microscope due to optical Bragg reflection [5].

A well-recognized challenge of working with BPLCs is their extremely narrow temperature ranges of <1 °C between the isotropic and cholesteric phases [9]. However, the operating temperature range for most of the LC-based devices, usually based on mixtures of different nematic LCs, is quite wide and normally centered around ambient temperature. Stabilization of BPs to exploit their useful properties is an ongoing and intense area of research in the liquid crystal community. Blue phases have been stabilized by polymer templating, bent-core mesogens, bimesogens and the addition of nanoparticles [10]. Polymer stabilization can increase the temperature range of BP by about an order of magnitude [11]. The monomers crosslink inside the BP defects and stabilize the BPLC by introducing an elastic interaction and removing the volume and free energy of the disclination lines [12]. Chiral dopants with high twisting power are used to increase the elastic chirality driving forces. Achiral bent core dopants, known to display chiral conformations and create chiral environments, are more effective than chiral dopants since they both increase the chiral elastic forces and reduce, due to their shape, the free energy in the vicinity of the defects [10].

Nanoparticles are less thermodynamically stable as compared to microparticles and tend to aggregate in the high energy defects of liquid crystals. The well understood topological defects generated by colloidal microspheres in LCs have been exploited to scaffold plasmonic gold nanoparticles. The nanoparticles locate inside the defects and show shape-dependent self-orientation with respect to the microsphere surface [13]. Whereas LC defects form on the surface of micron-size particles, nanoparticles, closer in size to the mesogens, can change the mesophase properties by affecting phase transition temperatures and the order parameter, similar to a molecular additive. Functionalizing the nanoparticles with mesogenic ligands is seen as an effective approach to reduce the disturbance of the liquid crystal order. Nanoparticle stabilization is explained by their incorporation into the BP disclination lines to reduce the overall LC free energy by removing elastic deformation energy within the defects [9,14,15]. Dierking et al. found 100% defect occupation and widening of temperature range for the 40 nm silica nanospheres [10].

In general, it is very challenging to produce stable LC dispersions of AuNRs due to their stronger tendency to aggregate through lateral interactions as compared to spherical NPs, along with the expulsion of particles by the LC elastic forces. However, AuNRs offer size asymmetry, distinctive surface electromagnetic fields and novel optical effects in plasmonics. As a polymer ligand for NPs, poly (ethylene oxide) (PEO), also called poly (ethylene glycol) (PEG), offers the advantage of solubility in water and organic solvents and, in the case of hydrogen bonded LCs, can act as a hydrogen bond acceptor. Smalyukh and co-workers have successfully produced stable dispersions of PEGylated AuNRs in nematic LCs [16,17]. Wong et al. reported an AuNR dispersed BP system: the BPLC became saturated above 0.004 wt% AuNRs with a maximum increase in the BP temperature range of 3 deg [18]. Functionalizing the nanoparticles with mesogenic ligands is seen as an effective approach to reduce the disturbance of the liquid crystal order. Wang et al. dispersed AuNRs functionalized with mesogenic ligands in a commercial BP mixture and found a widening of ΔT_BP_ from ~5.5 to a maximum of ~9 °C with a doping level of 0.07 wt% above which ΔT_BP_ decreased [19]. Nanorods and nanotubes of the optimal dimensions, i.e., diameters smaller than the defect cores (~10 nm) and lengths on order of the BP lattice size (~300–400 nm), should result in more efficient BP stabilization because they should not only fill the singular defect sites but also the disclination lines across several unit cells [10].

Theoretical works also suggest that BPLCs are effective templates of micron-sized colloids to form tunable photonic crystals [20,21]. A new application of BPLCs as templates to control the spatial distribution of nanomaterials was demonstrated by Gharbi et al., who studied gold spherical nanoparticles functionalized with mesogenic ligands in BPLCs [22]. The 4.5 nm NPs assemble into giant cubic lattices by selectively migrating into periodic strong trapping sites in the BP disclination lines. These giant NP lattices are matched to BP structures with similar lattice parameters. At the phase transition of BP I to BP II, the NP lattice reversibly switches between two different cubic structures. This finding is of significance since the simultaneous presence of two different structures in the same material may have potential applications as dynamic optical materials. Even with very high loadings of gold NPs of up to 25 wt%, the BP lattice parameters remained the same. Similar to other gold NP/BPLC studies, the gold NPs only increased the cyanobiphenol-based BP mixture temperature range from ΔT ~0.9 to 2.5 °C and did not perturb the BP lattice.

**Figure 3:**
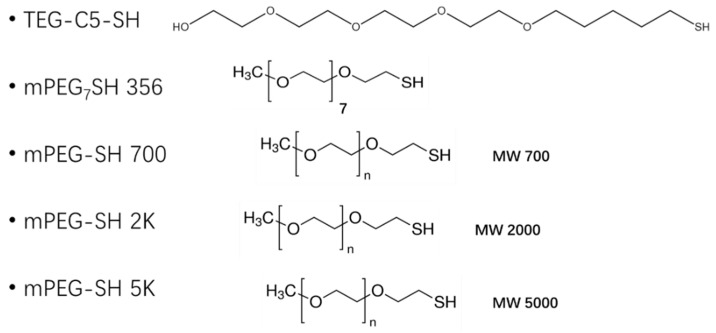
The abbreviation of the ligands and their structures.

Here, ethylene oxide oligomers and polymers with structures shown in Figure 3 and functionalized gold nanoparticles were dispersed in a BPLC mixture based on the mesogenic acid heterodimers, n-hexylbenzoic acid (6BA) and n-*trans*-butylcyclohexanecarboxylic acid (4-BCHA) with the chiral dopant (R)-2-octyl 4-[4-(hexyloxy)benzoyloxy]benzoate [23]. This particular BP mixture was chosen due to its relatively low and wide temperature range since thiol functionalized gold NPs will decompose at higher temperatures. Furthermore, unlike the previously reported wide range BP mixture that used n-*trans*-hexylcyclohexanecarboxylic acid instead of 6BA, the 4-BCHA-6BA mixture showed no large hysteresis, that is, the BP temperature range was the same in the heating versus cooling cycles [24].

To compare the effect of adding PEO alone versus tethered to the AuNPs and AuNRs, different MW PEO oligomers were added to the optimized BP mixture. The choice of very short chain PEO (M_n_ ~300 to 400), essentially oligomers, is based on a study by Dierking who added short chain polystyrene to a cholesteryl-based BP mixture and found that the lowest molecular weight used, M_n_ = 490, gave the maximum BP range [11]. Thiolated PEO ligands of the same molecular weights were synthesized and used to prepare the AuNP-PEO, where the gold cores have an average diameter of ~4.7 nm, and the AuNRs-PEO, where the gold mini rods have dimensions of 57 × 7.6 and 33 × 6.3 nm. The polymer functionalized mini-AuNRs are assumed to concentrate inside the disclination lines and decrease free energy by taking up the defect volumes. Since the mini-AuNRs have widths smaller than the theoretical disclination line ~10 nm diameter, the BP phase structure may be better conserved. The effect of the molecular weight and concentration of the added guest component on the BP temperature range and phases was studied by polarized optical microscopy (POM).

## 2. Materials and Methods

### 2.1. Materials

Hexadecyltrimethylammonium bromide (CTAB) (≥99%), gold (III) chloride trihydrate (HAuCl_4_·3H_2_O) (≥99.9%), sodium borohydride (NaBH_4_) (≥99%), silver nitrate (AgNO_3_) (99+%) and hydroquinone (≥99%) were purchased from Sigma-Aldrich (Oakville, ON, Canada). Trans-4-n-Butylcyclohexanecarboxylic acid (4-BCHA) was purchased from Alfa Aesar (Tewksbury, MA, USA). Sodium hydroxide (NaOH) was purchased from Fisher Scientific (Ottawa, ON, Canada), 4-Hexylbenzoic acid (6BA), (S)-octan-2-yl 4-((4-(hexyloxy)benzoyl)oxy)benzoate (chiral dopant), methoxy polyethylene glycol thiol (mPEG-SH) with a molecular weight of 5, 2, 700 and 356 K were previously synthesized. The synthesis of 6BA is reported in a previous publication [25]. 4-Dimethylaminopyridine (DMAP) stabilized gold nanoparticles were prepared following the procedure by Rucarneau et al. [26]. The synthesis method of mini-AuNRs nanorods with tunable plasmonic peaks beyond 1000 nm was carried out according to the procedure developed by Murphy and coworkers [27].

### 2.2. Functionalization of Gold Nanoparticles

Gold nanospheres: In a typical synthesis, 125 mL of PEG-SH solution (0.45 $\times 10^{-3}$ M in 95% ethanol or Milli-Q water) was added to a 125 mL aqueous solution of DMAP–Au NP. The clear, burgundy red mixture was allowed to stand at room temperature overnight. Neither changes in color nor precipitation were observed. The clear burgundy solution was rotary evaporated and the reddish solid was taken up in a minimum volume of water, transferred to a dialysis bag and dialyzed against water, over 5 days, to remove any unbound PEG ligands [28].

***Mini-AuNRs:*** Ligand solutions in Milli-Q water of around 30 mg/mL were previously prepared. The concentration of the stock mini-AuNR-CTAB solution was 0.6572 g/L. Volumes of mini-AuNRs were diluted with 1 mL Milli-Q water and were added dropwise into the ligand solutions at a rate of one drop/60 s. For the different ligands, these volumes were 500 (TEG-C5-SH), 200 (mPEG_7_-SH), 150 (mPEG-SH 2K) and 200 μL (mPEG-SH 5K). The ratio between the ligands and gold atoms was around 100. After adding the AuNRs, the solutions were left stirring at 27 °C for 24 h for complete exchange. The functionalized mini-AuNRs were then washed with Milli-Q water and centrifuged three times. After ligand exchange, mini-AuNRs were dissolved in Milli-Q water for preservation. Complete exchange and removal of the CTAB by this procedure was verified in earlier work using energy dispersive spectroscopy (EDS) and X-ray photoelectron spectroscopy (XPS) [29]. The concentrations of AuNRs were calculated based on the UV–vis absorbance values, NR size and the extinction coefficients as outlined by Murphy and coworkers [27]. The TEM analysis of the size and shapes of the nanorods are given in the Supplementary Material.

### 2.3. Preparation of BPLC Mixture

Pure blue phase mixtures were prepared by mixing 31.2% of 4-Hexylbenzoic acid (6BA), 32.5% of *trans*-4-Butylcyclohexanecarboxylic Acid (4-BCHA) and 36.2% chiral dopant in 2 mL tetrahydrofuran (THF) (Fisher Scientific, Ottawa, ON, Canada). The mixtures were then heated at 70 °C under vacuum and argon atmosphere to evaporate THF solvent overnight. The chemical structures and corresponding contents are shown in Table 1. It was determined that when the chiral dopant is 36% and 6BA and 4-BCHA are a 1:1 mixture, the resulting blue phase is the most stable with a temperature range of ~6.5 °C both during cooling and heating process, that is, no supercooling occurred.

### 2.4. Preparation of the Blue Phase Nanoparticle Dispersions

The required amount of functionalized AuNPs and AuNRs were added in the BP mixture as a solution in THF. The AuNRs-BP mixtures were thoroughly mixed by vortexing vigorously followed by sonicating for 1 min. The solvent was then evaporated under a slow flow of inert gas at 50 °C overnight. Finally, the samples were vacuum dried at the same temperature for 1 h to eliminate any residual solvent. The exact composition of these mixtures was calculated based on integrated peaks of ^1^H NMR of the mixtures.

### 2.5. Characterization

Optical microscopy. All glass slides were treated with aqua regia and rinsed with water, acetone and hexane before use to completely remove any surface anchoring effects. An Eclipse LV100POL optical microscope (Nikon, Tokyo, Japan) with 5× magnification was used with a Mettler FP52 heating plate.

A certain amount of blue phase mixture was taken out of the glass vial and dissolved in THF. A weighted amount of PEO, that was dried in a vacuum chamber over two days, was added and the components were further mixed by sonication. A drop of these homogenous solutions was placed on a preheated glass slide. After 45–60 min, a cover slide was placed on top. The sample was then imaged in the polarized mode.

UV–vis spectroscopy. The UV–vis spectra were acquired using a Cary 5000 UV-VIS-NIR spectrophotometer (Agilent Technologies, Santa Clara, CA, USA) which has photometric performance in the 175–3300 nm range.

Transmission Electron Microscopy (TEM). TEM images of mini-AuNRs were acquired using a Philips CM200 200 kV TEM (Philips, Eindhoven, The Netherlands) with an AMT XR40B CCD Camera and an EDAX Genesis Analysis System. The samples were diluted in Milli-Q water and deposited on a carbon copper grids (CF 400-Cu) that were dried under vacuum overnight.

## 3. Results

### 3.1. Stabilization of BP Mixture by Short Chain PEO

Polymer stabilization of blue phases normally consists of adding a reactive mesogen (monomer) to the blue phase. The monomers migrate into the lattice of disclination lines and in situ polymerization is carried out, usually by UV irradiation. Dierking and others have pointed out that the very large temperature ranges reported for the polymer stabilized BP samples, as well as for dimesogens and bent-core LCs, are often the result of supercooling. In fact, the BP phase is often only observed upon cooling [10]. Kasch et al. showed that considerable thermodynamically stable widening of the BP temperature range of a cholesteryl nonanoate and benzoate mixture could be achieved by simply adding short chain polystyrene (PS). Unlike the in situ polymerization, the molecular weight was well defined. A blue phase stability range of up to 12K, one of the largest measured on heating, that varied smoothly as a function of polymer volume fraction, was reported. When the guest molecule (polymer) concentration is just below the solubility limit, microscale phase separation begins. As the polymer begins to fill up the high energy defects, the BP range dramatically increases. Interestingly, short chain poly (methyl methacrylate), PMMA, produced negligible widening, showing that molecular interactions must be taken into account [11].

**Figure 4:**
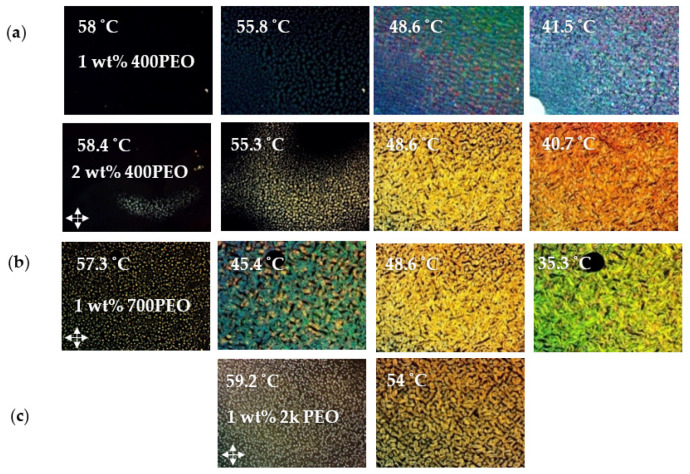
POM images of the cooling cycles of (**a**) 1 and 2 wt% PEO 400, (**b**) 1 wt% PEO 700 and (**c**) 1 wt% PEO 2000 dispersed in the BP mixture. The nucleation of the cholesteric phase for 1 wt% PEO 400 at 41.5 °C is indicated by the bright white spot in the bottom left side corner of the image.

Table 2 lists the BP temperature ranges for the 1:1 6BA:4CA with 36 wt% chiral dopant BP mixture (BPM36) with PEO oligomers of different molecular weights and concentrations. Representative POM images for the samples are presented in Figure 4. PEO molecular weights of 300–400, 700 and 2000 g/mol were studied. The blue phase was only observed for the lowest concentration, 1 wt%, of the lowest molecular weight oligomer, PEO 300. The BP range upon cooling increased from ΔT_BP_ = 6.5 to 13.1 °C but the heating cycle, ΔT_BP_ = 5.5 °C, basically remained the same. Similar to the study conducted by Kasch et al., higher molecular weights were less effective and only cholesteric phases were observed for PEO 700 and 2000. For concentrations as low as 0.5 wt%, phase separation is evident at the transition from the isotropic to the cholesteric phase. Kasch et al. found that the BP stabilization was only effective for the short chain PS with molecular weights lower than that of the cholesteryl mesogens [11]. In general, only very low molecular weight flexible polymers (oligomers) are miscible with liquid crystals. Highly flexible polymers such as PEO or polystyrene (PS) will phase separate at the isotropic-LC transition due to the entropic cost of transforming from a random coil to an extended chain compatible with the mesophase orientational order [30,31,32]. Additionally, polymers can lead to an increase in the thermal hysteresis of the blue phase [33].

### 3.2. Stabilization of BP Mixture by AuNP-PEO

Stabilization of BPs by nanoparticles is generally much less effective than polymer stabilization. In a notable exception, Cordoyiannis et al. reported the selective widening of BP III by over 20 °C by adding hydrophobic surface treated CdSe NPs [34]. Other than phase stabilization, there are two other major motivations to combine BP LCs with nanoparticles. Although BPs have an ultrafast response to applied electric fields, the required voltages are prohibitively high for applications, and this is even a worse issue for polymer stabilized BPs. However, polymer stabilized BPs doped with NPs showed electro-optical switching with low voltages, no hysteresis and fast response speeds [35,36,37]. The second motivation for combining NPs and BPs is for templating purposes, as demonstrated by the assembly of gold NPs with mesogenic ligands into giant cubic lattices [22]. Given the synthetic effort to produce highly specific mesogenic ligands to chemically match the ligand shell with the LC matrix, polymer functionalized NPs present a simpler alternative, plus may provide additional BP stabilization.

**Figure 5:**
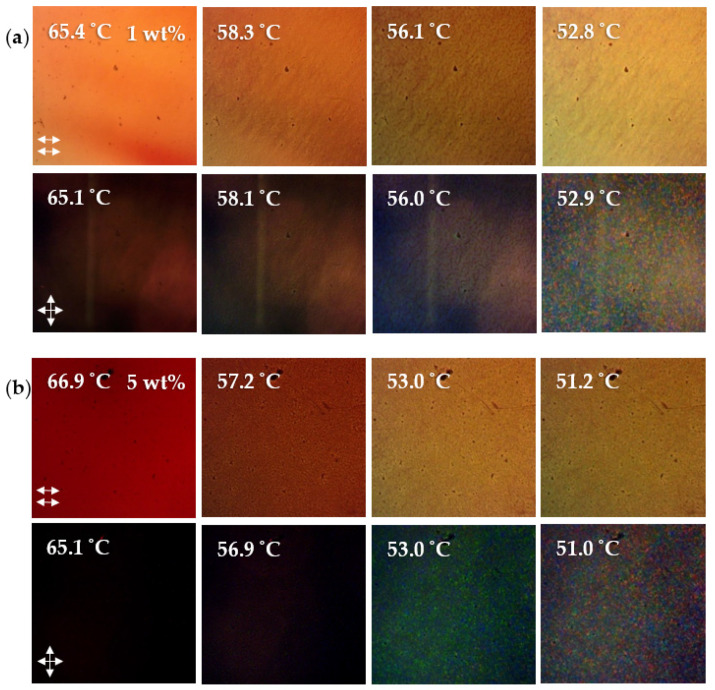
POM images of cooling cycles for (**a**) 1 and (**b**) 5 wt% of AuNP-300PEO in BPM36 under parallel and crossed polars.

**Figure 6:**
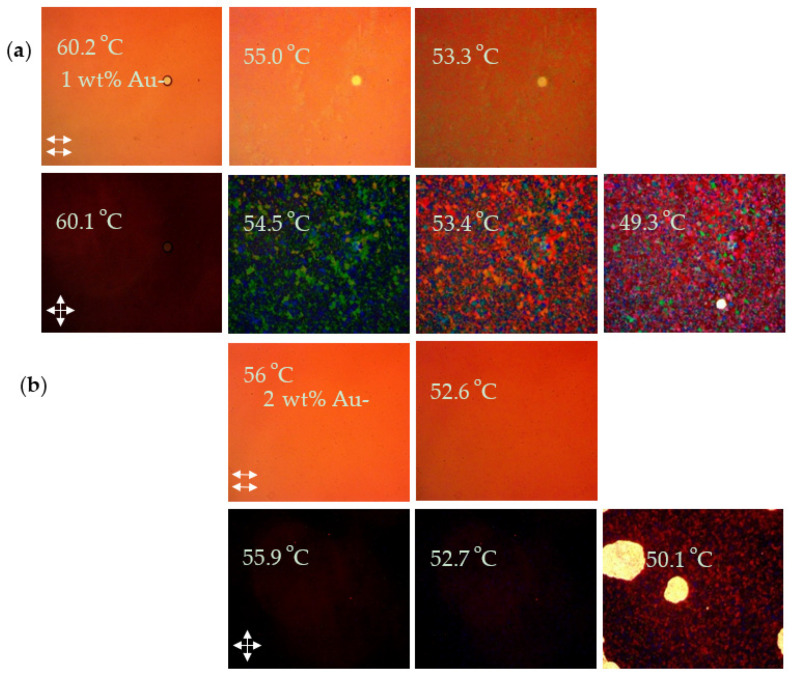
POM images of cooling cycles for (**a**) 1 and (**b**) 2 wt% AuNP-PEO 700 in BPM36 under parallel and crossed polars.

Table 3 presents the BP temperature ranges for the 6BA:4CA BP mixtures with AuNP-PEO of different PEO molecular weights and concentrations. Representative POM images for the samples are presented in Figure 5 and Figure 6. In contrast to the free PEO, the AuNP-PEO can be added up to a much higher concentration before phase separation and/or disappearance of the BP occurs. The guest species tend to migrate into the cores of the BP disclination lines where the LC molecules are disordered, presumably until the total volume of these defects is filled. Given the high density of gold (19.3 g/cc) as compared to PEO (1.13 g/cc), it is not surprising that the BP can accommodate a much higher loading of AuNP-PEOs by weight. For AuNP-PEO300 and AuNP-PEO700, micron size aggregates are not detected by POM until above 10 and 2 wt% loadings, respectively, above which only the cholesteric phase is observed. We would expect the maximum loading of the AuNP-PEO to match that of the free PEO in terms of the PEO content. Given that the organic content of the AuNP-PEO300 as measured by TGA is 18 wt%, the 10 wt% NP loading corresponds to ~1.8 wt% PEO.

### 3.3. Stabilization of 6BA:4-BCHA BP Mixture by AuNR-PEO

**Figure 7:**
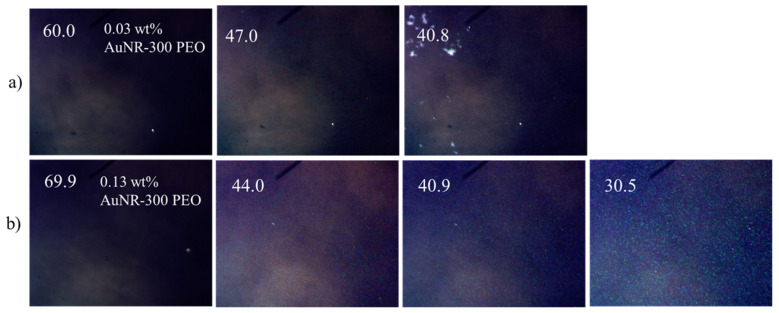
POM images under crossed polars of (**a**) 0.03 and (**b**) 0.13 wt% AuNR-300 PEO in BPM36.

**Figure 8:**
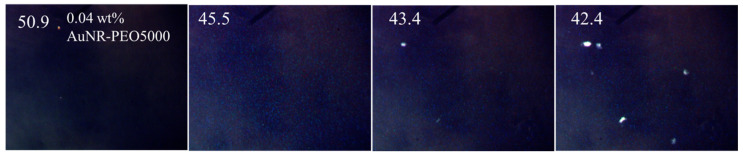
POM images under crossed polars of 0.04 wt% AuNR-5000 PEO in BPM36.

The effects of gold nanorods (AuNRs) with different ligands on the miscibility and stability of BP were separately tested using three ethylene oxide-based ligands: PEO 5K, PEO 300 and PEO 2K. The ligand PEO 5K on short mini-AuNRs (size: 33.5 × 6.3 nm) was also tested and two samples with different mass percentages were compared. For the longer mini-AuNRs (size: 57.2 × 7.5 nm), all four ligands’ functionalities were prepared and tested. The results are summarized in Table 4. Some representative POM images are showed in Figure 7 and Figure 8. In general, the polymer functionalized AuNRs that formed stable dispersions in isotropic solvents behaved like the short chain polymers. Among those, AuNR-PEO300 with 0.13 wt% shows the largest increase in the BP temperature range to ΔT_BP_ > 13.5 °C. We were not able to determine the lower end of T_BP_ since our microscope is only equipped with heating and not cooling below ambient temperature. The highest molecular weight ligands (mPEG 2K and 5K) had a small effect, only slightly increasing the BP range. With the monodispersed AuNRs, the AuNRs-BPLC composites form a bicontinuous system where the LC mixture can form the BP structure and also disperse the AuNRs-PEO. The reproducibility of the effect of AuNRs on the BP temperature range was checked in the case of the mPEG-SH 2K ligand (Table 5). There was some variation of the BP temperature ranges and the transition temperatures among the different 0.03 wt% AuNR-PEO2000 samples, perhaps due to mass weighing errors given the very small quantities. These samples also showed some AuNR aggregates co-existing with the BP, indicating that the sample is saturated even at this low doping level.

## 4. Discussion

The AuNP-PEO nanospheres had little effect on the BP range, which was also observed in the case of the same size AuNPs with mesogenic ligands [22]. We propose that the AuNPs do not simply fill in the disclination lines as previously depicted in many schematics of NP stabilized BP studies in the literature. For example, see reference [14]. Instead, as shown by small angle X-ray scattering (SAXS), the AuNPs with mesogenic ligands selectively locate at strong specific trapping sites within the lattice of BP disclination lines to form different cubic NP lattices for BP I versus BP II [22]. These sites are remarkably robust given that the same Bragg reflections for each phase appear independent of NP concentration or whether the sample is cooled or heated. Whether this also is occurring for the AuNP-PEO, which is made from the same precursor AuNPs, is worth investigating. Whereas guest species such as polymers or small molecules can completely fill the disclination lines, the specific site trapping in the case of AuNPs and perhaps for other spherical NPs, in general, may explain their minimal effect on the BP range.

It is interesting to compare the effect of the AuNRs on the BP stability with doping by the same polymers, free and tethered to gold nanospheres. In this study, only the gold core size and shape were changed from the previous study, yet the results are drastically different. The anisotropic shape of the AuNRs may better stabilize the BP by filling in the disclination lines instead of becoming trapped into specific sites. Although only the AuNRs of two sizes functionalized with PEG 5K were compared, the data indicate that the longer mini-AuNRs better stabilized the BP. The short mini-AuNRs, closer to a spherical shape, may be more easily trapped in specific sites, whereas the elongated shape of the long mini-AuNRs can more efficiently fill the disclination lines.

In contrast to the gold nanospheres, the effect of the molecular weight of the mPEG ligands of the AuNRs on the BP stability did not follow an obvious trend since the AuNR-PEO 2K and AuNR-PEO 5K also stabilized the BP to some extent, whereas the spherical AuNPs functionalized with the same ligands totally suppressed the BP. This diminished influence of the ligand molecular weight is expected since the polymer ligand shell makes up a much smaller volume fraction of the AuNRs as compared to the 4 nm AuNPs. In general, the PEGylation of CTAB stabilized AuNRs by exchange results in very low grafting densities (<0.05 chains/nm^2^) [38], whereas the PEG grafting densities on the spherical AuNPs are very high (>4 chains/nm^2^) [26]. Overall, the lowering of the free energy due to the more effective defect core replacement by the AuNRs is large enough to counter the entropic effect from the polymer ligands. In addition, aggregates were observed to coexist with the BP in the case of the AuNRs-PEG 2K, indicating that the excess AuNRs-PEG 2K are totally phase separated and do not influence the BP formation.

## 5. Conclusions

The results presented here demonstrate that polymer functionalized AuNRs are a promising additive to both stabilize and provide additional functionality to blue phase liquid crystals. Further experiments and characterization based on the results described above should be pursued. As with the vast majority, if not all, of the other AuNR/LC studies in the literature, the particle concentration is calculated by adding the amount of known concentrations of AuNRs and LC and assuming no loss of material, which is not realistic given the very small quantities that are normally used. The synthesis of larger volume samples and an analytical method to measure the actual gold mass needs to be carried out to accurately determine the relationship between the AuNR concentration and ΔT_BP_. Secondly, the heating versus cooling process can cause different kinetics of the phase transitions of the blue phase. The free energy goes into any local minimum during the heating process, while the cooling process will preserve the energy minimum of BP II [39]. As mentioned previously, the BP mixture used here, pure or doped with mPEG and AuNPs, did not show any such thermal hysteresis in contrast to the BP mixture originally reported in the literature. However, heating cycles of AuNRs-BP composites with a microscope equipped with cooling below ambient temperature should also be carried out to rule out any supercooling. The variation of the temperature ranges of the AuNRs-BP observed here can partially be due to different levels of spherical particle impurities in AuNRs samples that are quite difficult to completely remove. Given that spherical nanoparticles have little effect on ΔT_BP_, the presence of these impurities may reduce the stabilization of the BP by AuNRs. Therefore, inorganic nanorods or nanotubes that are quite uniform and monodispersed and can be synthesized in much larger quantities will be tested, such as TiO_2_ nanorods functionalized with PEO ligands. Such polymer functionalized nanorods, also known to form lyotropic LC phases in organic solvents, may fill the disclination lines more efficiently.

## Figures and Tables

**Table 1:** Chemicals, their structures and mass percentage used in forming blue phase mixture.

Chemicals	Structure	Mass Percentage (%)
4-hexylbenzoic acid (6BA)	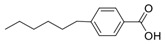	31.2
*trans*-4-butylcyclohexanecarboxylic acid (4BCHA)	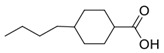	32.5
(S)-octan-2-yl 4-((4-(hexyloxy)benzoyl)oxy)benzoate (chiral dopant)	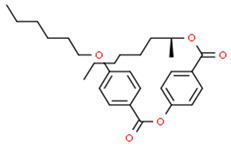	36.2

**Table 2:** Transition temperatures and the mesophase ranges for the BP-PEO mixtures.

BP Mixture		Observed Phases	BP Temperature Ranges, °C	
			Cooling (ΔT_BP_)	Heating (ΔT_BP_)
1:1 6BA:4CA 36 %wt chiral dopant (BPM36)		BP I, II and III, Ch	54.5–61 (6.5)	57.5–60 (6.5)
BPM36	1 wt% PEO400	BP I, II and III, Ch	57.6–44.4 (13.1)	52.5–58 (5.5)
BPM36	2 wt% PEO400	Ch	53	
BPM36	1 wt% PEO700	Ch	57.9	

**Table 3:** Transition temperatures and mesophase ranges for the BP-AuNP PEO mixtures.

BP Mixture wt% (vol%) AuNP		Observed Phases	BP Temperature Ranges	
			Cooling (ΔT_BP_)	Heating (ΔT_BP_)
BMP36		BP I, II and III, Ch	54.5–61 (6.5)	57.5–60.6 (7.1)
BMP36	1 wt% (0.39 vol%) Au-PEO300	BP I, II and III, Ch	52.5–58.5 (6)	56.9–60 (3.1)
BMP36	5 wt% (2.0 vol%) Au-PEO300	BP I, II and III, Ch	50.8–57.8 (7)	51.7–58 (6.3)
BMP36	10 wt% (4.2 vol%) Au-PEO300	Ch	56	
BMP36	1 wt% (2.4 vol%) Au-PEO700	BP I, II and III, Ch	50.5–57.5 (7)	
BMP36	2 wt% (4.79 vol%) Au-PEO700	BP I, II and III, Ch	51–53.5 (2.5)	
BM36	5 wt% (11.49 vol%) Au-PEO700	Ch	52.8	

**Table 4:** Temperature range and mass percentage of gold in BP of short mini-AuNR (33.5× 6.3 nm) BP composites with ligands of mPEG-SH 5K.

Ligands	Pure BP Temperature Range	Mass Percentage of Gold in BP	Temperature Range
mPEG-SH 5K	54.8–60.7 °C (5.9 °C)	0.0692%	No blue phase formed
		0.0374%	51.0–57.2 °C (6.2 °C)

**Table 5:** Phases observed for BP mixture and AuNRs-BP composites of long (57.2 × 7.5 nm) mini-AuNRs.

BP Mixture	wt% AuNR	Observed Phases	BP Temperature Ranges Cooling (ΔT_BP_)
BMP36		BP I, II and III, Ch	48.5–55.8 (7.3)
BMP36	0.13 wt% AuNR-PEO300	BP I, II and III, Ch	<30.5–44.0 (>13.5)
BMP36	0.03 wt% AuNR-PEO300	BP I, II and III, Ch	40.8–49.7 (8.9)
BMP36	0.03 wt% AuNR-PEO2000	BP I, II and III, Ch	47.0–56.3 (9.3) 30.7–42.6 (11.9) 37.3–47.0 (9.7)
BMP36	0.07 wt% AuNR-PEO2000	BP I, II and III, Ch	31.1–40.6 (8.5)
BMP36	0.04 wt% AuNR-PEO5000	BP I, II and III, Ch	42.4–50.0 (7.6)

## Data Availability

Data is contained within the article or Supplementary Material.

## Supplementary Materials

The following are available online at https://www.mdpi.com/article/10.3390/nano12010091/s1, Figure S1: Histograms of the lengths and widths of short mini-AuNRs, Figure S2: TEM images of short mini AuNRs functionalized with different ligands, Figure S3: Histograms of the lengths and widths of long mini-AuNRs Figure S4: TEM images of long mini AuNRs functionalized with different ligands: Table S1 Shape, size and quantity of short mini-AuNRs functionalized with different ligands., Table S2: Shape, size and quantity of long mini-AuNRs functionalized with different ligands.

## References

[1] Johnson D., Flack J., Crooker P.P. (1980). Structure and properties of the cholesteric blue phases. Phys. Rev. Lett..

[2] Meiboom S., Sethna J.P., Anderson P.W., Brinkman W.F. (1981). Theory of the blue phase of cholesteric liquid crystals. Phys. Rev. Lett..

[3] Kitzerow H.S. (2009). Blue phases come of age: A review. Emerg. Liq. Cryst. Technol. IV.

[4] Rahman M.A., Said S.M., Balamurugan S. (2015). Blue phase liquid crystal: Strategies for phase stabilization and device development. Sci. Technol. Adv. Mater..

[5] Fukuda J.I., Žumer S. (2010). Novel Defect Structures in a Strongly Confined Liquid-Crystalline Blue Phase. Phys. Rev. Lett..

[6] Bahr C., Kitzerow H.S. (2001). Chirality in Liquid Crystals.

[7] Kikuchi H. (2007). Liquid Crystalline Blue Phases. Liquid Crystalline Blue Phases in Liquid Crystalline Functional Assemblies and Their Supramolecular Structures.

[8] Yoshizawa A. (2013). Material design for blue phase liquid crystals and their electro-optical effects. RSC Adv..

[9] Ravnik M., Alexander G.P., Yeomans J.M., Zumer S. (2010). Mesoscopic modelling of colloids in chiral nematics. Faraday Discuss..

[10] Dierking I., Blenkhorn W., Credland E., Drake W., Kociuruba R., Kayser B., Michael T. (2012). Stabilising liquid crystalline blue phases. Soft Matter.

[11] Kasch N., Dierking I., Turner M. (2013). Stabilization of the liquid crystalline blue phase by the addition of short-chain polystyrene. Soft Matter.

[12] Jo S.Y., Jeon S.W., Kim B.C., Bae J.H., Araoka F., Choi S.W. (2017). Polymer stabilization of liquid-crystal blue phase II toward photonic crystals. ACS Appl. Mater. Interfaces.

[13] Senyuk B., Evans J.S., Ackerman P., Lee T., Manna P., Vigderman L., Zubarev E.R., van de Lagemaat J., Smalyukh I.I. (2012). Shape-dependent oriented trapping and scaffolding of plasmonic nanoparticles by topological defects for self-assembly of colloidal dimers in liquid crystals. Nano Lett..

[14] Karatairi E., Rožič B., Kutnjak Z., Tzitzios V., Nounesis G., Cordoyiannis G., Thoen J., Glorieux C., Kralj S. (2010). Nanoparticle-induced widening of the temperature range of liquid-crystalline blue phases. Phys. Rev. E.

[15] Yoshida H., Tanaka Y., Kawamoto K., Kubo H., Tsuda T., Fujii A., Kuwabata S., Kikuchi H., Ozaki M. (2009). Nanoparticle-stabilized cholesteric blue phases. Appl. Phys. Exp..

[16] Senyuk B., Glugla D., Smalyukh I.I. (2013). Rotational and translational diffusion of anisotropic gold nanoparticles in liquid crystals controlled by varying surface anchoring. Phys. Rev. E.

[17] Liu Q., Tang J., Zhang Y., Martinez A., Wang S., He S., White T.J., Smalyukh I.I. (2014). Shape-dependent dispersion and alignment of nonaggregating plasmonic gold nanoparticles in lyotropic and thermotropic liquid crystals. Phys. Rev. E.

[18] Wong J.M., Hwang J.Y., Chien L.C. (2011). Electrically reconfigurable and thermally sensitive optical properties of gold nanorods dispersed liquid crystal blue phase. Soft Matter.

[19] Wang L., Gutierrez-Cuevas K.S., Krishna Bisoyi H., Xiang J., Singh G., Zola R.S., Kumar S., Lavrentovich O.D., Urbas A., Li Q. (2015). NIR Light-directing self-organized 3D photonic superstructures loaded with anisotropic plasmonic hybrid nanorods. Chem. Commun..

[20] Ravnik M., Alexander G.P., Yeomans J.M., Žumer S. (2011). Three-dimensional colloidal crystals in liquid crystalline blue phases. Proc. Natl. Acad. Sci. USA.

[21] Stratford K., Henrich O., Lintuvuori J.S., Cates M.E., Marenduzzo D. (2014). Self-assembly of colloid-cholesteric composites provides a possible route to switchable optical materials. Nat. Commun..

[22] Gharbi M.A., Manet S., Lhermitte J., Brown S., Milette J., Toader V., Sutton M., Reven L. (2016). Reversible nanoparticle cubic lattices in blue phase liquid crystals. ACS Nano.

[23] Roohnikan M., Cummings Premack K., Guzman-Juarez B., Toader V., Rey A., Reven L. (2019). Hydrogen-bonded LC nanocomposites: Characterization of nanoparticle-LC interactions by solid-state NMR and FTIR spectroscopies. Liq. Cryst..

[24] Gvozdovskyy I. (2015). ‘Blue phases’ of highly chiral thermotropic liquid crystals with a wide range of near-room temperature. Liq. Cryst..

[25] Roohnikan M., Toader V., Rey A., Reven L. (2016). Hydrogen-bonded liquid crystal nanocomposites. Langmuir.

[26] Rucareanu S., Maccarini M., Shepherd J.L., Lennox R.B. (2008). Polymer-capped gold nanoparticles by ligand-exchange reactions. J. Mater. Chem..

[27] Chang H.H., Murphy C.J. (2018). Mini gold nanorods with tunable plasmonic peaks beyond 1000 nm. Chem. Mater..

[28] Corbierre M.K., Cameron N.S., Sutton M., Laaziri K., Lennox R. (2005). Gold nanoparticle/polymer nanocomposites: Dispersion of nanoparticles as a function of capping agent molecular weight and grafting density. Langmuir.

[29] Ni S. (2021). Gold Nanocomposites: Synthesis and Applications. Ph.D. Dissertation.

[30] Matsuyama A., Kato T. (1999). Phase separations and orientational ordering of polymers in liquid crystal solvents. Phys. Rev. E.

[31] Matsuyama A., Kato T. (2000). Induced nematic phase in a polymer/liquid crystal mixture. J. Chem. Phys..

[32] Matsuyama A. (2003). Conformational transitions of a semiflexible polymer in nematic solvents. Phys. Rev. E.

[33] Chen K.M., Gauza S., Xianyu H., Wu S.T. (2010). Hysteresis effects in blue-phase liquid crystals. J. Display Technol..

[34] Cordoyiannis G., Losada-Pérez P., Tripathi C.S.P., Rozic B., Tkalec U., Tzitzios V., Karatairi E., Nounesis G., Kutnjak Z., Musevic I. (2010). Blue phase III widening in CE6-dispersed surface-functionalised CdSe nanoparticles. Liq. Cryst..

[35] Wang L., He W., Wang Q., Yu M., Xiao X., Zhang Y., Ellahi M., Zhao D., Yang H., Guo L. (2013). Polymer-stabilized nanoparticle-enriched blue phase liquid crystals. J. Mater. Chem. C.

[36] Wang L., He W., Xiao X., Meng F., Zhang Y., Yang P., Wang L., Xiao J., Yang H., Lu Y. (2012). Hysteresis-free blue phase liquid-crystal-stabilized by ZnS nanoparticles. Small.

[37] Choudhary A., Singh G., Biradar A.M. (2014). Advances in gold nanoparticle–liquid crystal composites. Nanoscale.

[38] Schulz F., Friedrich W., Hoppe K., Vossmeyer T., Weller H., Lange H. (2016). Effective PEGylation of gold nanorods. Nanoscale.

[39] Oton E., Netter E., Nakano T., D-Katayama Y., Inoue F. (2017). Monodomain blue phase liquid crystal layers for phase modulation. Sci. Rep..

