# Supplementary material for: Polymer Functionalized Nanoparticles in Blue Phase LC: Effect of Particle Shape
Source: Nanomaterials (Basel). 2021 Dec 29;12(1):91. doi: 10.3390/nano12010091 (PMC8746361; doi:10.3390/nano12010091)
Supplement: Supplementary file 1 [file nanomaterials-12-00091-s001.zip › nanomaterials-1466881-supplementary.pdf]

nanomaterials

Supplementary Information

Polymer Functionalized Nanoparticles in Blue Phase LC: Effect of Particle Shape

Manlin Zhang, Michael Lindner-D'Addario, Mahdi Roohnikan, Violeta Toader, Robert Bruce Lennox and Linda Reven *

Department of Chemistry, McGill University, Montreal, QC H3A 0B8, Canada; Manlin.Zhang@mail.mcgill.ca (M.Z.); michael.lindner-daddario@mail.mcgill.ca (M.L.-D.); Mahdi.Roohnikan@mail.mcgill.ca (M.R.); Violeta.Toader@mcgill.ca (V.T.); bruce.lennox@mcgill.ca (R.B.L.)
* Correspondence: linda.reven@mcgill.ca

Transmission Electron Microscopy (TEM) of the Gold Nanorods (AuNRs)

Table S1. Shape, size and quantity of short mini-AuNRs functionalized with different ligands.

Ligands	Rod Shape Size	Aspect Ratio	Quantity	Spherical Shape	Quantity
CTAB	34 nm ×6.3 nm	5.4	92%	14 nm	8%
TEG-C5-SH	34 nm ×6.3 nm	5.4			
mPEG-SH 5000	34 nm ×6.3 nm	5.4	92%	15 nm	8%

Table S2. Shape, size and quantity of long mini AuNRs functionalized with different ligands.

Ligands	Rod Shape Size	Aspect Ratio	Quantity	Spherical Shape	Quantity
CTAB	57 nm ×7.5 nm	7.6	91%	19 nm	9%
TEG-C5-SH	57 nm ×7.3 nm	7.8	96%	19 nm	4%
$mPEG_7SH$ 356	57 nm ×7.6 nm	7.5	96%	20 nm	4%
mPEG-SH 2000	57 nm ×7.5 nm	7.6	92%	19 nm	8%

(a)

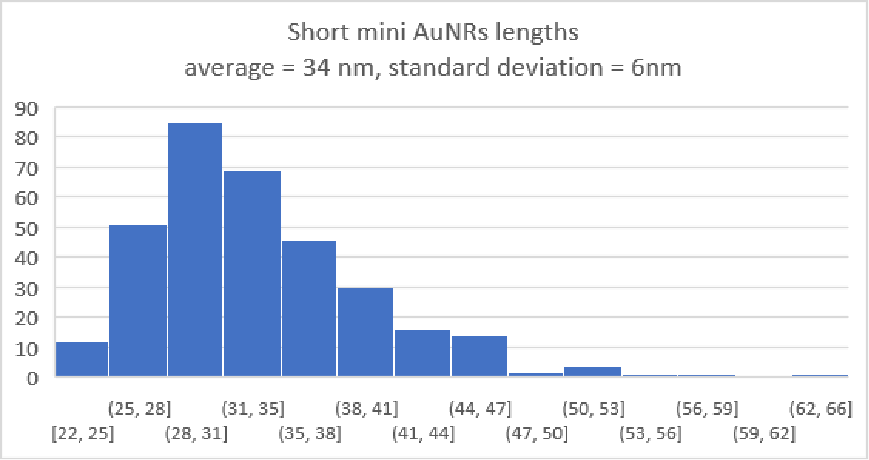

(b)

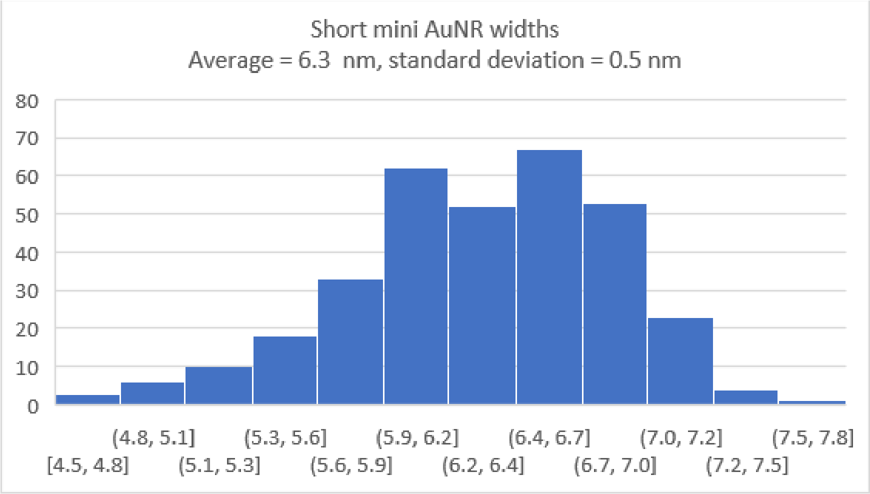

Figure S1. Histograms of the (**a**) lengths and (**b**) widths of short mini-AuNRs.

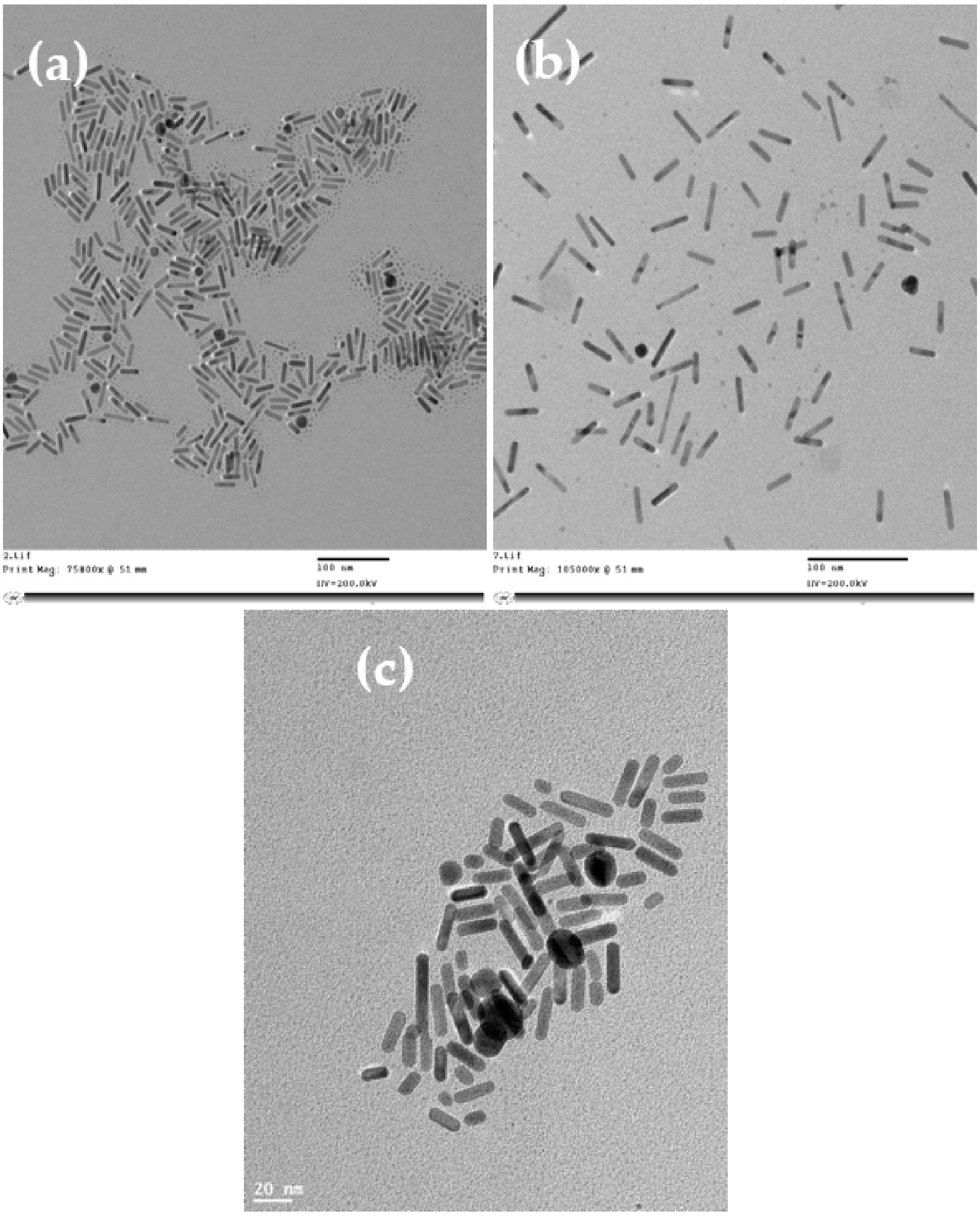

Figure S2. TEM images of short mini AuNRs functionalized with different ligands: (**a**) mini-AuNRs with CTAB, (**b**) mini-AuNRs with mPEG-SH 5K, (**c**) mini-AuNRs with TEG-C5-SH with some impurities.

(a)

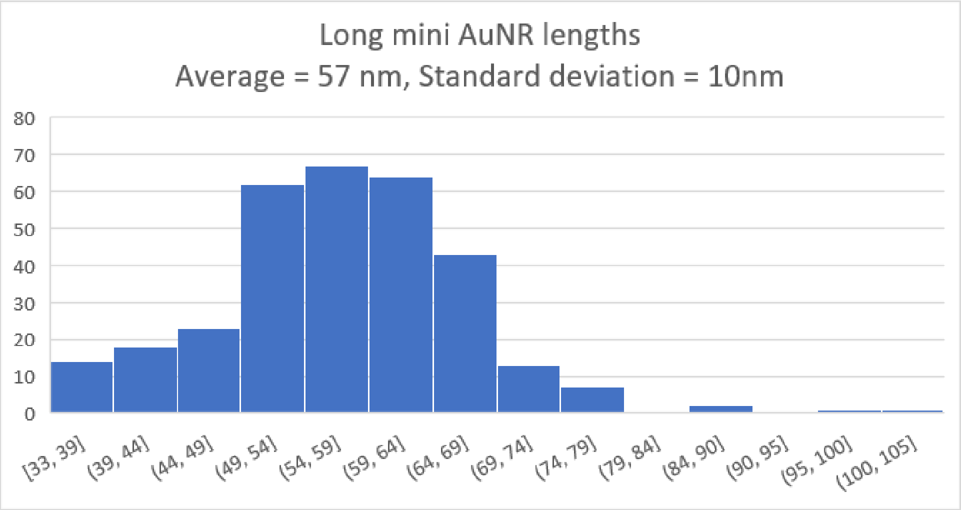

(b)

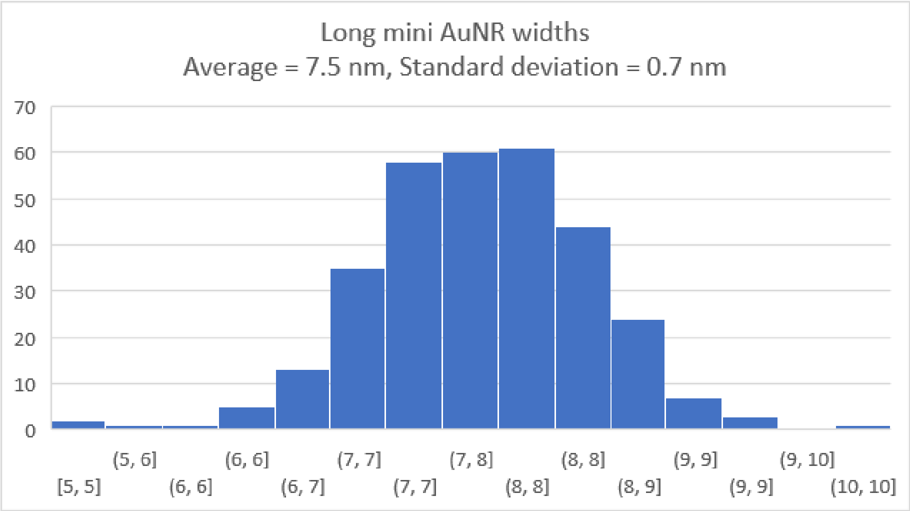

Figure S3. Histograms of the (**a**) lengths and (**b**) widths of long mini-AuNRs.

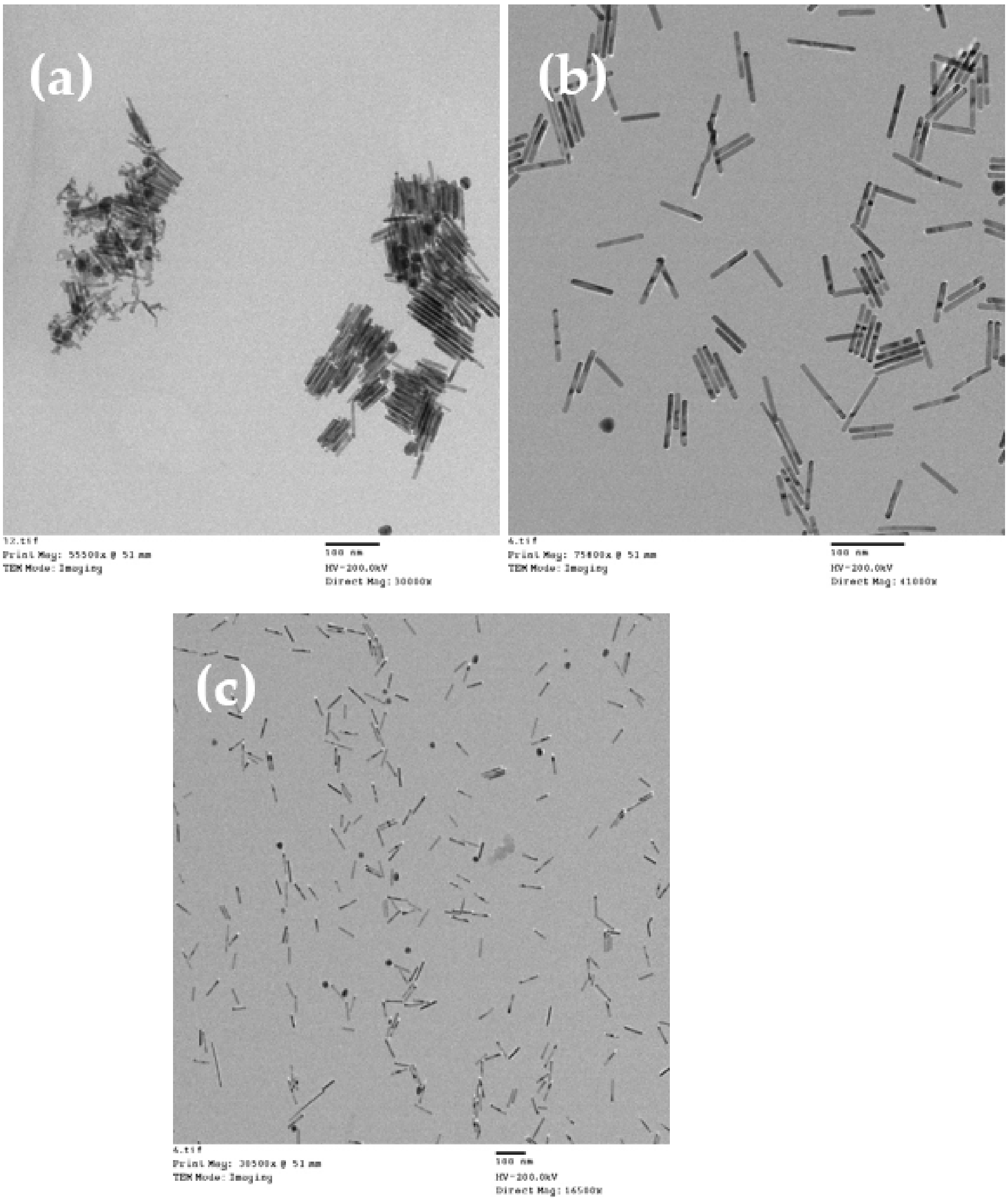

Figure S4. TEM images of long mini AuNRs functionalized with different ligands: (**a**) TEG-C5-SH, (**b**) mPEG$_7$SH 356, and (**c**) mPEG-SH 2K.
